# Prevalence and pattern of co morbidity among type2 diabetics attending urban primary healthcare centers at Bhubaneswar (India)

**DOI:** 10.1371/journal.pone.0181661

**Published:** 2017-08-25

**Authors:** Sandipana Pati, F. G. Schellevis

**Affiliations:** 1 Department of Health & Family Welfare, Government of Odisha, Bhubaneswar, Odisha, India; 2 NIVEL (Netherlands Institute for Health Services Research), Utrecht, The Netherlands, and Department of General Practice & Elderly Care Medicine, Amsterdam Public Health Research Institute,VU University Medical Center, Amsterdam, the Netherlands; University of Brescia, ITALY

## Abstract

**Objective:**

India has the second largest diabetic population in the world. The chronic nature of the disease and high prevalence of co-existing chronic medical conditions or “co morbidities” makes diabetes management complex for the patient and for health care providers. Hence a strong need was felt to explore the problem of co morbidity among diabetics and its dimensions in primary health care practices.

**Method:**

This cross sectional survey was carried out on 912 type 2 diabetes patients attending different urban primary health care facilities at Bhubaneswar. Data regarding existence of co morbidity and demographical details were elicited by a predesigned, pretested questionnaire“**Diabetes Co morbidity Evaluation Tool** in **Primary Care** (DCET- PC)”. Statistical analyses were done using STATA.

**Results:**

Overall 84% had one ormore than one comorbid condition. The most frequent co morbid conditions were hypertension [62%], acid peptic disease [28%], chronic back ache [22%] and osteoarthritis [21%]. The median number of co morbid conditions among both males and females is 2[IQR = 2]. The range of the number of co morbid conditions was wider among males [0–14] than females [0–6]. The number of co morbidities was highest in the age group > = 60 across both sexes. Most of the male patients below 40 years of age had either single [53%] or three co morbidities [11%] whereas among female patients of the same age group single [40%] or two co morbidities [22%] were more predominantly present. Age was found to be a strong independent predictor for diabetes co morbidity. The odds of having co morbidity among people above poverty line and schedule caste were found to be[OR = 3.50; 95%CI 1.85–6.62]and [OR = 2.46; CI 95%1.16–5.25] respectively. Odds were increased for retired status [OR = 1.21; 95% CI 1.01–3.91] and obesity [OR = 3.96; 95%CI 1.01–15.76].

**Conclusion:**

The results show a high prevalence of co morbidities in patients with type 2 diabetes attending urban primary health care facilities. Hypertension, acid peptic disease, chronic back ache and arthritis being the most common, strategies need to be designed taking into account the multiple demands of co morbidities.

## Introduction

Globally the burden of diabetes mellitus (DM) is a major public health concern. According to the estimates of DM burden worldwide, 371 million people actually have DM and about 80% live in Low and Middle Income countries. The number of people expected to have DM by 2030 is over 550 million. The number of people living with diabetes in India has increased from 61 million in 2011 to 67 million in 2014.[1] India has the second largest diabetic population after China in the world. Apart from being a chronic debilitating disease the high prevalence of co-existing chronic medical conditions or “co morbidities” make diabetes management an arduous task for the patient and for health care providers. Prior studies have proved that most adults with diabetes have at least one co morbid condition and 40%have three or more co morbid conditions yet the perspective of the healthcare providers and treatment strategies are more oriented on management of diabetes alone.[2–5]For optimal health care delivery and developing strategies that support self-management among the ever growing population of diabetes patients, we need to understand how the number, type, and severity of co morbidities influence these patients’ diabetes management. In an already burdened health care system co morbid conditions may shift the providers’ focus away from diabetes.[6,7] Co morbidities may also serve as competing demands on patients’ self-management resources, and potentially reduce the amount of time and energy left for diabetes self-care.[8–11] Even conditions not directly related to diabetes, such as pain and depression, are more prevalent in diabetics, thus emphasising the need to take into account both diabetes-related and non-diabetes related co morbidities.[7–9]

Studies carried out in various parts of India have mostly concentrated on single co morbidities like hypertension or depression.[12–16] Ramachandra et al in their study have focussed on the prevalence of micro and macro vascular complications among type 2 diabetics.[17]Yadav et al have studied the prevalence of hypertension and dyslipidemia among type 2 diabetics. Similarly various studies have studied the prevalence of dyslipidemia and hypertension among type 2 diabetics with focus on metabolic syndrome.[18–24] Further detailed study on the other possible co existing conditions will help the healthcare providers to be more observant and be prepared accordingly for the multiple demands of the co morbidities and the outcomes which can be extrapolated to other parts of the country especially eastern India. Therefore, we explored the presence and pattern of co morbidity and how the prevalence ofcomorbidity varies with patient characteristics among diabetes patients presenting to primary health care settings in Bhubaneswar, an urban area in the state of Odisha.

## Methodology

### Study design and setting

A cross sectional interview survey was conducted in all 17 urban primary health care centers in Bhubaneswar, the capital city of Odisha with a population of 900,000 inhabitants.[25]According to the National Sample Survey Office’s 71st round on social consumption of health about 72% of outpatient care in Odisha is provided by public health care professionals.[26]The public health care system has a three tier structure comprising of primary, secondary and tertiary levels. Primary Health Care Centres are involved in delivering primary care while district hospital and sub-divisional hospitals render secondary care. Tertiary health care is provided by medical college hospitals. Each primary healthcare center caters to a population of 30,000to40,000.

### Selection of study participants

Patients attending a primary health care center between September 2014 and February 2015, who had been diagnosed by a physician of having Type 2 diabetes mellitus (T2DM) for more than six months according to their personal medical record, were eligible to be included in the study. Since the consultation time is limited in the health centers and every interview took 20–30 minutes and there was only one interviewer available per center, for the feasibility of the study every third eligible type 2 diabetic patient was invited to participate. The inclusion criterium of a diabetes duration of six months was applied because we also collected information about health care utilisation for diabetes. Patients too ill to participate or with emergency health conditions, were excluded from the study. Anonymised details of all patients excluded (age, gender, reason for exclusion) were recorded in order to compare the characteristics of the participants with the non-participants. All patients were explained about the study purpose and written informed consent was obtained prior to the interview. To avoid duplication, every patient was given a unique code and any patient who had already been interviewed in any of the facilities previously was excluded.

#### Measurements

The participating patients were interviewed in a separate private chamber using a predesigned and pretested questionnaire–“**Diabetes Co morbidity Evaluation Tool** in **Primary Care**(DCET- PC)”.[S1 and S2 DCET-PC] The DCET-PC is derived from“Multimorbidity Assessment Questionnaire for Primary Care(MAQ-PC),”avalidatedquestionnaire [27]was pretested and the feedback was used to adapt the questionnaire for our study.

Two graduate nurses trained in patient history taking and interview techniques carried out the interviews and 10% of the interviews were carried out in the presence of first author.

The DCET- PC included questions about the existence of co morbid conditions, eliciting information on whether the patient had any of the listed chronic problems and socio-demographic details (age, sex, place of birth, residence, ethnicity (general, scheduled caste and tribe, other backward classes),religion, educational level, marital status, annual family income and ‘above poverty line’ or ‘below poverty line’ status of the household). An iterative process was followed to arrive at the list of most frequently occurring co morbidities to be included in the questionnaire. An extensive literature search, study of validated questionnaires along with a review of medical records of 200 diabetes patients were undertaken to prepare a first list of 14 conditions. The semi finalized list was then shared with a panel of sixprimary care physicians. They were requested to indicate the severity(marginal-very severe) and importance against each listed condition and also mention any additional diseases not mentioned in the list. The final list of 16 co morbid conditions was used in the questionnairre. The self reported conditions were ascertained by asking if it had been diagnosed by a doctor,and whether they were prescribed any medicines for the conditions.

The questionnaire was pretested on approximately 5% ofthe study sample i.e. 44 type 2 diabetes patients visiting non study centers. Only a few modifications were made in the questionnaire based on the feedback from pretesting the questionnaire. For measuring depression we had included the PHQ9,[28] however due to reluctance of the test respondents to answer these questions we omitted the PHQ9 and included a question about physician diagnosed depression. Similarly, as pretesting respondents found it too sensitive to report their monthly income we categorized income in the final version. We included “other” for any additional co morbidity that we might have missed in our list. Since many pretesting respondents could not recollect the date or month of the initial diagnosis of type 2 diabetes, we modified it to “year of diagnosis”. For the diabetics with stroke and aphasia it was decided to elicit data from the accompanying attendant and the answers were read aloud to the patient to check if he agreed.

Data entry was carried out by trained professionals. Random cross checking of 10% of data was done by the first author. From each center it was planned to select 50 patients making the total size 850. Before the study, we defined that we wanted to establish reliably a 10% prevalence of a co morbid condition, defined as a 95% Confidence Interval of 8–12%.

#### Analysis

Co morbidity prevalence was calculated in terms of the frequency of occurrence of each of the 16 chronic diseases. Descriptive results were expressed as means ± standard deviation or as number of participants and percentages. Bivariate comparisons were performed using Student’s t-test or one-way analysis of variance (ANOVA) for quantitative data and chi-square for categorical data. Diabetic patients were classified according to presence or absence of co morbidity (i.e. no versus one or more co morbid conditions). To examine how the prevalence of co morbidity varies with respect to age, gender, place of origin, socioeconomic status, a binary logistic model was used. To determine the independent association of patient characteristics with comorbiditya multivariate regression model was applied; we only considered variables showing a P value<0.05 in the univariate analysis. Statistical analyses were done using STATA.

### Ethical considerations

Respondents were informed about the objectives of the study and the use of information they would be giving and we collected their signature or thumb impression on the informed consent form. The data were coded and the identities of the respondents were kept completely confidential. The Odisha state research and ethics committee gave the ethical approval for the study (letter no. 161/SHRMU dt. 16.05.2014).

## Results

We approached 942 diabetics of which 912 consented to be interviewed [response rate of 97%]. The reasons cited for non-response were lack of time and unwillingness to answer. Of all respondents 575 [63%] were males. The highest number of respondents were in the age group of 40–69 years (N = 766 [83.4%]). Mean age of respondents was 55 years.

### Prevalence of co morbidity

**Fig 1:**
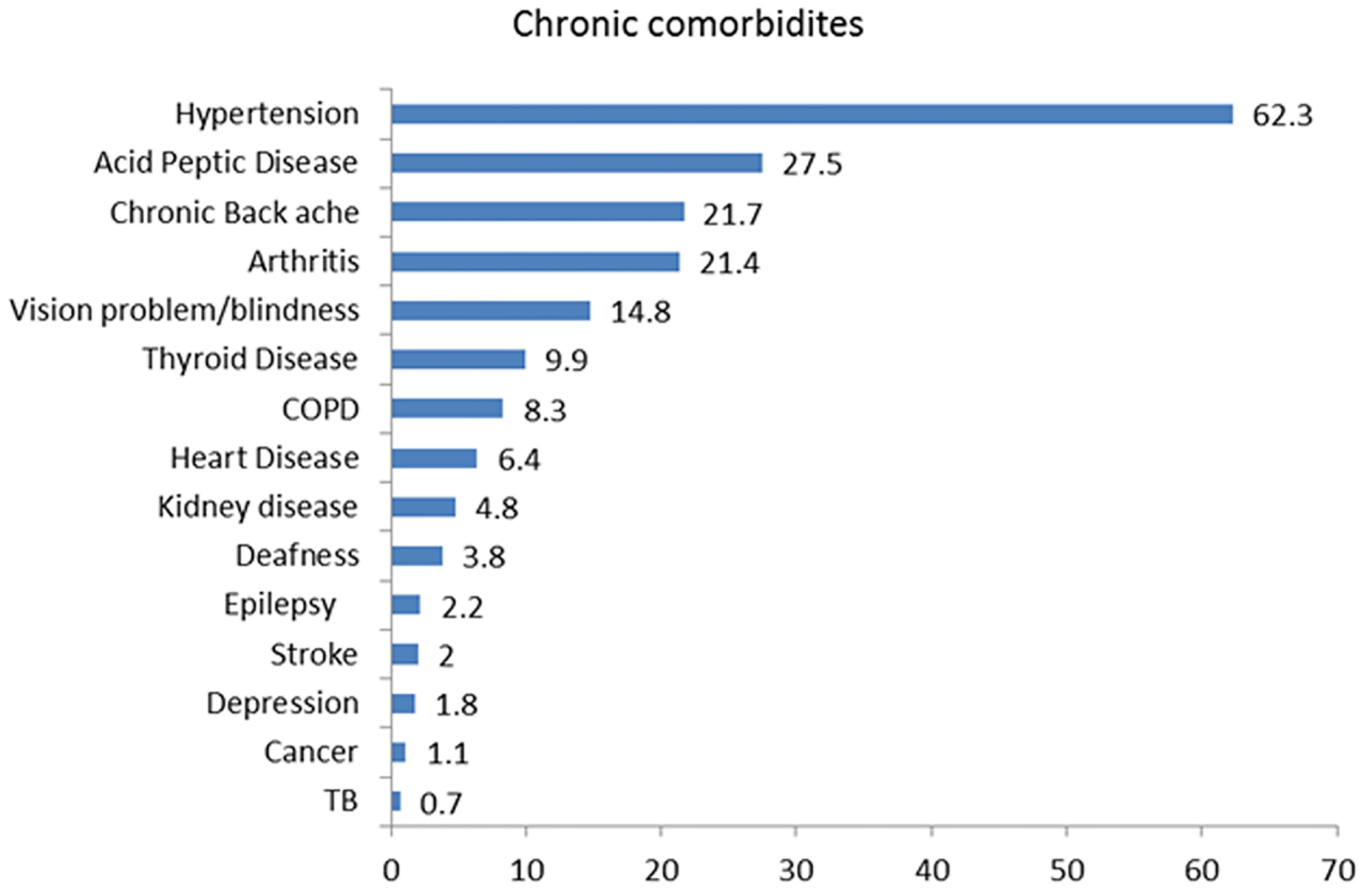
Prevalence rates of comorbid diseases among type 2 diabetes patients (N = 912; percentages).

The overall prevalence of co morbid conditions among the type 2 diabetic respondents was 84%. The most frequent co morbid conditions were hypertension [62%], acid peptic disease [28%], chronic back ache [22%] and osteoarthritis [21%] [Fig 1]

**Fig 2:**
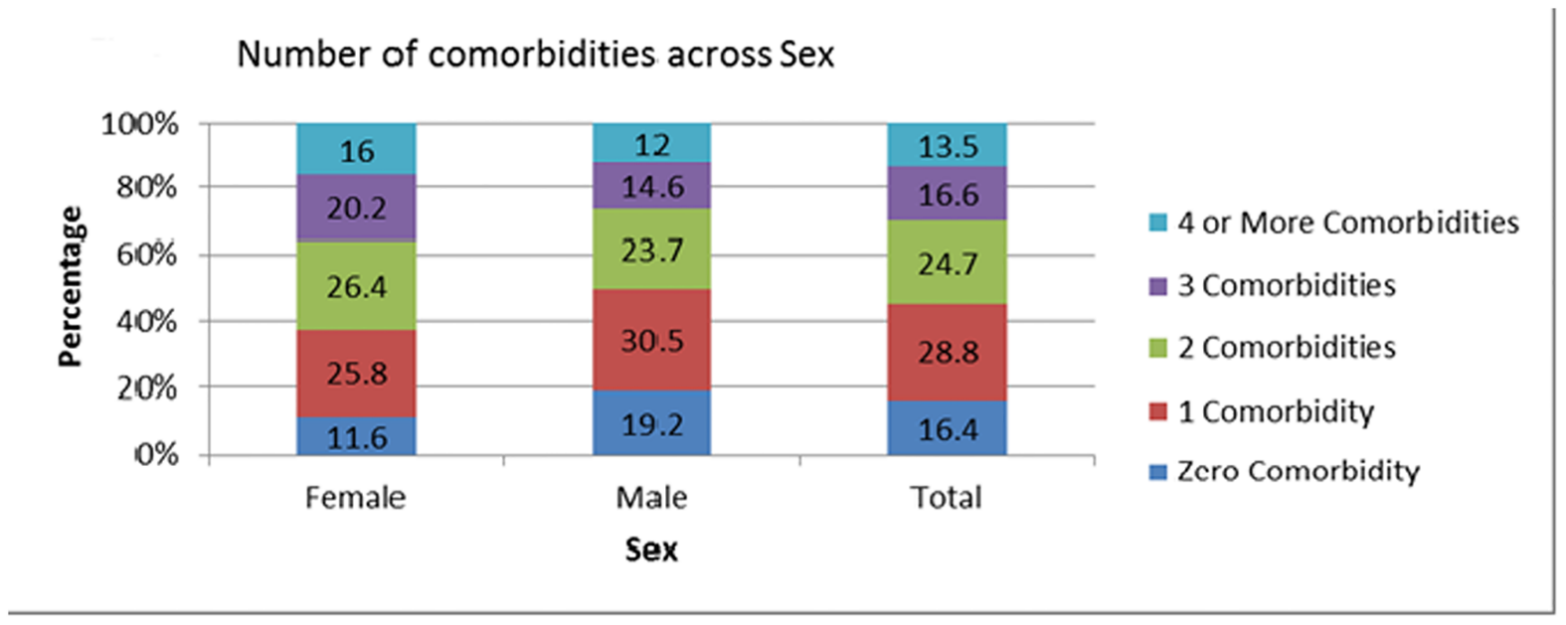
Number of comorbid diseases by sex among type 2 diabetes patients (N = 912; percentages).

Of all patients 16% had no co morbidity, 29% had a single co morbidity, 25% had two co morbidities and 30% were diagnosed with 3 or more co morbidities [Fig 2]. The median number of co morbid conditions among both males and females is 2[IQR = 2]. The range of the number of co morbid conditions was wider among males [0–14] than females [0–6]. The number of co morbidities was highest in the age group > = 60 across both sexes. Most of the male patients below 40 years of age had either single [53%] or three co morbidities [11%] whereas among female patients of the same age group single [40%] or two co morbidities [22%] were more predominantly present.

### Patient characteristics associated with co morbidity

Of 766 respondents having one or more co morbidities 637 (83%) were in the age group of 40–69 years. 61% of the respondents having co morbidities were males. Socioeconomically about 70% of diabetics with co morbidities were from a household above poverty line. [Table 1].

**Table 1:** Socio-demographic characteristics of type 2 diabetes patients, total and by comorbidity status (N = 912).

	Total(N = 912)	Co morbidity (N = 766) 84%	No Co morbidity (N = 146) 16%	p value (chi square)
**Age group (years)**				<0.001
18–29	0.3	0.1	1.3	
30–39	6.7	6.3	8.7	
40–49	21.7	20.3	28.7	
50–59	34.4	33.4	39.3	
60–69	27.5	29.5	17.3	
> = 70	9.5	10.5	4.7	
**Gender**				0.003
Male	63.1	61.0	74.0	
Female	36.9	39.0	26.0	
**Urbanisation level of residence**				0.143
Urban	78.0	78.4	76.0	
Semi Urban	10.9	11.4	8.7	
Rural	11.1	10.2	15.3	
**Ethnicity**				<0.001
Schedule Caste	28.8	31.5	14.7	
Schedule Tribe	11.8	13.1	5.3	
Other Backward Caste	14.2	12.6	22.7	
Others	45.2	42.8	57.3	
**Socio-economic status**				<0.001
Above Poverty Line	66.2	70.5	36.2	
Below Poverty Line	33.8	29.5	63.8	
**Highest Education**				0.332
Illiterate	8.4	8.4	8.7	
Primary	17.0	16.0	22.0	
Secondary	34.5	34.8	32.7	
University	40.2	40.8	36.7	
**Marital Status**				0.459
Unmarried	2.2	2.2	2.0	
Married	87.5	91.3	86.8	
Widower	9.5	10.0	6.6	
**Religion**				0.593
Hindu	88.9	88.4	92.0	
Muslim	6.9	7.3	4.7	
Christian	4.0	4.2	3.3	
Others	0.1	0.1	0.0	
**Employment**				0.002
Employed	45.3	42.8	58.0	
Unemployed	9.0	10.1	3.3	
Homemaker	27.6	28.8	21.3	
Retired	18.2	18.3	17.3	
**Family history of diabetes**				<0.001
Yes	22.4	24.7	10.7	
No	77.6	75.3	89.3	
**Risk Factor: Body Mass Index**				<0.001
Underweight	2.5	2.1	4.7	
Normal	23.3	20.0	40.0	
Overweight	19.4	19.4	19.3	
Obese	54.8	58.5	36.0	

Age was found to be a strong independent predictor for diabetes co morbidity [41-59yr: OR = 2.12; 0.95–4.73 &> = 60 yrs. OR = 6.08; 2.13–17.34]. The odds of having co morbidity among people above poverty line and schedule caste were found to be 3.50[1.85–6.62] and2.46[1.16–5.25] respectively(compared to below poverty line and general caste{caste not included in schedule caste, schedule tribe or other backward caste}). Retired status [OR = 1.21; 95% CI 1.01–3.91] and Obesity [OR = 3.96; 95%CI 1.01–15.76] had increased odds of having a co morbidity after adjusting for other variables. [Table 2]

**Table 2:** Patient characteristics associated with comorbidity in patients with type 2 diabetes mellitus (N = 912).

	OR [95% CI]	Adjusted OR [95%CI]
***Age group (years)***		
< = 40	reference	reference
41–59	1.29 [0.74–2.24]	**2.12 [0.95–4.73]**
> = 60	2.93 [1.58–5.44]	**6.08 [2.13–17.34]**
***Gender***		
Male	reference	reference
Female	1.82 [1.23–2.70]	0.89 [0.40–2.01]
***Urbanisation level of residence***		
Rural	reference	reference
Semi Urban	1.97 [0.93–4.15]	2.08 [0.69–6.28]
Urban	1.54 [0.93–2.57]	1.37 [0.61–3.07]
***Ethnicity***		
General	reference	reference
Schedule Caste	2.88 [1.75–4.72]	**2.46 [1.16–5.25]**
Schedule Tribe	3.28 [1.54–7.01]	**2.74 [1.06–7.06]**
Other Backward Class (OBC)	0.74 [0.47–1.17]	0.89 [0.41–1.90]
***Socio—economic status***		
Below Poverty Line	reference	reference
Above Poverty Line	2.11 [1.45–3.07]	**3.50 [1.85–6.62]**
***Highest education***		
Illiterate	reference	--
Primary	0.75 [0.37–1.52]	--
Secondary	1.10 [0.56–2.15]	***--***
University	1.15 [0.59–2.23]	--
***Marital status***		
Single	reference	--
Married	0.62 [0.34–1.14]	--
***Religion***		
Non-Hindu	reference	--
Hindu	1.51 [0.80–2.84]	--
***Employment***		
Employed	reference	reference
Unemployed/Home maker	2.13 [1.41–3.23]	1.84 [0.75–4.52]
Retired	1.43 [0.88–2.31]	**1.21 [1.01–3.91]**
***Family history of diabetes***		
No	reference	reference
Yes	2.762 [1.60–4.76]	1.65 [0.77–3.54]
***Risk factor*: *BMI***		
Underweight	reference	reference
Normal	1.16 [0.43–2.84]	1.32 [0.34–5.13]
Overweight	2.23 [0.84–5.90]	3.47 [0.78–15.29]
Obese	3.62 [1.42–9.19]	**3.96 [1.01–15.76]**

## Discussion

In this study, we found that the vast majority (84%)of the patients with diagnosed type 2 diabetics in a primary care population in Bhubaneswar had at least one chronic co morbid condition, 25% had two co morbid conditions and 30% were diagnosed with 3 or more co morbid conditions. The most frequent co morbid chronic conditions were hypertension [more than half of the patients], acid peptic disease, chronic back ache and osteoarthritis each in about a quarter of the patients. The mean number of co morbid conditions was higher among females than males, whereas the range of the number of co morbid conditions was wider among males than females. The number of co morbidities was highest in the age group > = 60 and increased age was found to be a strong predictor for diabetes co morbidity. The odds of having co morbidity among people from a household above poverty line and schedule caste were found to be higher. Being retired and obese resulted in increased odds of having co morbidity.

The prevalence of co morbidity in patients with diagnosed T2D in this study was similar to or higher than those of previous studies.[29–32]. As observed in previous studies our study has also found the highest rate of co morbidity in the elderly, and a strong positive association with increasing age.[33,34] Banjareand Pradhan in their study on multimorbidity in the Bargarh district of Odisha have also reported that the multimorbidity rate among elderly increases with age.[35] Pati et al in their study on prevalence of multimorbidity in Odisha have also reported that chances of multimorbidity are higher among older age groups and higher socioeconomic groups.[36]In high income countries, persons with low socio economic status are more likely to have a higher number of co morbid conditions as compared to people from higher socio economic strata.[37,38] In contrast to these findings from western countries, we found a positive association between income and co morbidity in our population. Similar observations have been reported from other low and middle income countries.[33,39]. Lack of accessible health care for the lower income group could be a reason for less diagnoses of co morbid conditions among them. The present study has identified hypertension, acid peptic disease, chronic back ache and arthritis as the most common comorbid conditions among diabetics and these findings are in line with previous studies carried out in other parts of the world.[40–43] Yadav et al, Patel et al and Yadav et al in their respective studies conducted among type 2 diabetics in India have also reported high rates prevalence of hypertension.[18,44,45] Our study findings are also in line with prior studies that found a strong association between diabetes and gastrointestinal symptoms like gastroesophageal reflux and acid peptic disease.[42,46]

The major strength of this study is the primary care representativeness, which allows for generalization of the findings to primary care patients in India. We elicited information on 16 co morbid conditions among diabetics which were selected through an iterative process. Our study covers all adult age groups and is representative of users of primary health care facilities in terms of sex, age group, ethnicity and othersocio economic factors. To our knowledge, this is the first study to explore the magnitude of co morbidity among diabetics in a large and representative primary care sample in Odisha, India.

There are several limitations to this study. Our study population consisted only of diagnosed type 2 diabetics, the absence of undiagnosed diabetics is a limitation. Previous studies have stated that almost half of the diabetes cases remained undiagnosed till there is manifestation of some complication.[17,47,48] Prevalence rates of co morbidity among the undiagnosed diabetes patients will therefore be lower. Our study being a cross-sectional study limits the establishment of causal inference to socio economic factors and co morbidities. Secondly, self-reported chronic disease status is subject to self-declaration bias due to under-reporting of diagnosis or forgetfulness.[49,50] Incomplete diagnosis of physicians, lack of standardised criteria of diagnosis, missing validity checks of the diagnosis are among the other limitations.

Our study findings indicate a high prevalence of co morbidities among type 2 diabetes patients and hence multiple demands on the primary health care providers. Health care providers caring for T2DM patients should take co morbid conditions into account since co morbidity is the rule rather than the exception. Different co morbid conditions managed separately by different health care professionals might lead to the risk of fragmented care among the co morbidity patients. Our observations underline the importance for formulation of coordinated and comprehensive primary health care policies for clinical care of diabetics which includes not only diabetes care, but also care for the most common co morbid conditions. It also underlines the need to raise the competence level of primary health care providers to address these demands. Our findings support policies to strengthen generalistic primary health care. Our study results suggest the relevance of further studies on the impact of co morbidity on healthcare outcomes including healthcare utilisation and quality of life. Also further analysis of different combinations of co morbidity could shed light on a possible common etiology, and then on possibly preventive measures.

## Supporting information

S1 DCET-PC Questionnaire in English. (PDF)

S2 DCET-PC Questionnaire in Odia. (PDF)
