# Supplementary material for: Prevalence and pattern of co morbidity among type2 diabetics attending urban primary healthcare centers at Bhubaneswar (India)
Source: PLoS One. 2017 Aug 25;12(8):e0181661. doi: 10.1371/journal.pone.0181661 (PMC5571911; doi:10.1371/journal.pone.0181661)
Supplement: S1 DCET-PC — (PDF) [file pone.0181661.s001.PDF]

DCET-PC Questionnaire

1. Date:
2. Code No........
3. Age (in years).......
4. Sex.....................
5. Completed education... [_] Illiterate [_]Primary level [_]High School or Secondary level [_] Graduation or above
6. Religion [_]Hindu [_] Muslim[_]Christian [_]Others
7. Marital Status [_]Unmarried [_]Married [_}Widow[_]Separated
8. Ethnicity [_]SC [_]ST [_]OBC[_] Others
9. No. of people living in your household (including you)...........
10. Employment status-[_] Employed[_]Unemployed[_]Homemaker[_]Retired
11. On an average how many hours do you work in a day ....(hrs)
12. Net monthly income..[_] <10k [_] 10k-20k [_] 20k-30k [_] >30kINR
13. Net monthly income of your household ....INR
14. APL/BPL (as per ration card)..................................
15. Housing type [_]Kutcha [_]Pucca [_]Semi Pucca
16. Place of living [_] Urban [_] Semi Urban [_]Rural
17. Where have you spent major part of your adult life [_] Urban [_] Semi Urban [_] Rural
18. Family history of diabetes (parents or siblings suffer from diabetes) [_] Yes [_] No
19. Date on which you were diagnosed with diabetes mellitus __/_ _/ _ _
20. Where were you first diagnosed with diabetes [_]Public health care facility [_] Private health care facility
21. Presently are you taking any prescribed medicines for diabetes [_]Yes [_]No.
22. If yes, are you taking [_]Oral antidiabetes pills[_] Insulin [_]Both
23. Are you taking any alternative medicines for diabetes [_]Ayurvedic [_]Homeopathic [_]Others [_]None

24.Co existing Conditions		Yes/ No
**Arthritis**	A. Have you ever been **diagnosed** by a doctor with ***Arthritis****?*	Yes[_] No[_]
	In the **last 12 months** have you experienced ***pain, aching, stiffness or swelling in or around the joints (like arms, hands, legs or feet)*** which were not related to an injury and lasted for more than a month?	Yes[_] No[_]
	If yes, then are you taking any prescribed medications?	Yes[_] No[_]
**Hypertension**	Have you ever been **diagnosed** by a doctor with ***high blood pressure***?	Yes[_] No[_]
	If Yes, then are you taking any prescribed medicines for high blood pressure (hypertension)?	Yes[_]No[_]
	Not Applicable	NA
**Chronic Lung Diseases(Including Asthma)**	Have you ever been **diagnosed** with ***Chronic Lung Disease (Emphysema, Bronchitis, Asthma, COPD)?***	Yes[_]No[_]
	If yes, then are you taking any prescribed medications for it?	Yes[_]No[_]
	Not Applicable	NA
**Acid Peptic Disease**	Have you ever been **diagnosed** by a doctor with ***Acid –Peptic Ulcer disease*** (Gastritis) in the **last 12 months**?	Yes[_] No[_]
	Not Applicable	NA
**Chronic Back ache**	In the last 12 months have you been **diagnosed** by a doctor with ***chronic back pain***?	Yes[_] No[_]
	In the **last 12 months,** have you had continuous Back pain for more than 3 weeks?	Yes[_] No[_]
**Heart disease**	Have you ever been **diagnosed** by a doctor with ***Angina/heart attack/heart disease?***	Yes[_] No[_]
	If yes then are you taking any prescribed medicines for it?	Yes[_] No[_]
	In the **last 12 months** have you experienced ***any pain or discomfort*** in your chest when you walk uphill or hurry or normal walking?	Yes[_] No[_]

**Stroke**	Have you ever **been told** by a health professional that you have had a ***Stroke***?	Yes[_] No[_]
	If yes, are you taking any prescribed medication for it?	Yes[_] No[_]
	In the **last 12 months** have you suffered from **sudden onset of paralysis or weakness** in your arms or legs on one side of your body for **more than 24 hours?**	Yes[_] No[_]
**Blindness**	Have you been diagnosed by a doctor with blindness?	Yes[_] No[_]
	Do you have ***difficulty with vision*** (Answer **No** if you can see **OK** with **glasses**)?	Yes[_] No[_]
**Deafness**	In the **last 12 months** have you been diagnosed by a doctor with **deafness**?	Yes[_] No[_]
	In the **last 12 months** do you have ***Deafness or difficulty in hearing*** for **more than 3 months?**	Yes[_] No[_]
**Cancer**	Have you ever been **diagnosed** by a doctor with any type of **cancer**?	Yes[_] No[_]
**Chronic Kidney Diseases**	Have you ever been **diagnosed** by a doctor with ***long term kidney problem***?	Yes[_] No[_]
	Have you ever been on dialysis?	Yes[_] No[_]
**Epilepsy**	Have you ever **been told** by a health professional that you have ***Epilepsy***?	Yes[_] No[_]
	If yes, are you taking any prescribed medications?	Yes[_] No[_]
	Have you ever **suffered** from ***sudden onset of seizure*** *while at work or at rest*?	Yes[_] No[_]
**Thyroid Disease**	Have you ever been **diagnosed** by a doctor with ***Thyroid diseases***?	Yes[_] No[_]
	If yes are you taking any prescribed medication?	Yes[_] No[_]
**Tuberculosis**	Do you suffer from ***TB***?	Yes[_] No[_]
	Are you **under** any **treatment for *TB***?	Yes[_] No[_]
**Depression**	Have you ever been **diagnosed** by a doctor with **depression**?	Yes[_]No[_]
	In the last twelve months have you consulted a doctor for **feeling sad or depressed, worried or anxiety**?	Yes[_]No[_]
	If yes, are you taking any prescribed medications for depression?	Yes[_]No[_]
	Not Applicable	NA

25. Do you suffer from any other chronic health problems? [_] Yes [_] No

If yes, name them-

1...

-Is it diagnosed by a doctor? Yes [_]No[_]

-Are you taking any prescribed medications for it? Yes [_]No[_]

-How much is it limiting you in your daily life? 1. Not at all [_] 2.A little [_] 3.Somewhat [_]

4. Quite a bit [_] 5. A lot [_]

2...

-Is it diagnosed by a doctor? Yes[_]No[_]

-Are you taking any prescribed medications for it? Yes[_]No[_]

-How much is it limiting you in your daily life? 1. Not at all [_] 2.A little [_] 3.Somewhat [_]

4. Quite a bit [_] 5. A lot [_]

26. How much do you spend on medications per month?

1. For diabetes-...............INR

2. For other diseases.............INR

27. How much money do you spend on travel expenses for every visit to the health care facility..............INR

28. How many times in the last 6 months have you visited a health care facility? ....

29. How much money on an average do you spend in 6 months on laboratory investigations and tests for diabetes (blood sugar test, glycated haemoglobin estimation)? ....INR

-How much time do you spend on these tests? ....

30. How much money do you spend on laboratory tests for other chronic conditions?......................INR

-How much time do you spend for these tests?......................................

31. Height.........................

Weight..................

BMI............................
>25 Yes[_] No[_]
<25 Yes[_] No[_]
