# Supplementary material for: Prevalence and pattern of co morbidity among type2 diabetics attending urban primary healthcare centers at Bhubaneswar (India)
Source: PLoS One. 2017 Aug 25;12(8):e0181661. doi: 10.1371/journal.pone.0181661 (PMC5571911; doi:10.1371/journal.pone.0181661)
Supplement: S2 DCET-PC — (PDF) [file pone.0181661.s002.pdf]

ପ୍ରଶ୍ନାବଳୀ

ଏହି ଅଧ୍ୟୟନରେ ଭାଗ ନେବା ପାଇଁ ସମ୍ମତି ପ୍ରଦାନ କରିଥିବାରୁ ଏବଂ ଆପଣଙ୍କର ମୂଲ୍ୟବାନ ସମୟ ଦେଇଥିବାରୁ ଧନ୍ୟବାଦ । ଆପଣ ପ୍ରଦାନ କରିଥିବା ଏହି ସୂଚନା କଡାକଡି ଭାବରେ ଗୋପନୀୟ ରଖାଯିବ ଏବଂ କେବଳ ଗବେଷଣା ଅଧ୍ୟୟନ ଉଦ୍ଦେଶ୍ୟରେ ବ୍ୟବହାର କରାଯିବ ।

1. ତାରିଖ

2. କୋଡ୍ ନମ୍ବର........

3. ବୟସ ବର୍ଷରେ.......

4. ଲିଙ୍ଗ....................

5. ସମ୍ପୂର୍ଣ୍ଣ କରିଥିବା ଶିକ୍ଷାଗତ ଯୋଗ୍ୟତା... [_] ନିରକ୍ଷର [_]ପ୍ରାଥମିକ ସ୍ତର [_]ହାଇସ୍କୁଲ ବା ସେକେଣ୍ଡାରୀ ସ୍ତର [_] ସ୍ନାତକ ବା ଅଧିକ

6. ଧର୍ମ [_]ହିନ୍ଦୁ [_] ମୁସଲମାନ[_]ଖ୍ରୀଷ୍ଟିଆନ୍ [_]ଅନ୍ୟାନ୍ୟ

7. ବୈବାହିକ ସ୍ଥିତି [_]ଅବିବାହିତ [_]ବିବାହିତ [_]ବିଧବା[_]ପୃଥକ ହୋଇଥିବା

8. ବର୍ଣ୍ଣ [_]ଅନୁସୂଚିତ ଜାତି [_]ଅନୁସୂଚିତ ଜନଜାତି [_]ଅନ୍ୟାନ୍ୟ ପଛୁଆବର୍ଗ[_] ଅନ୍ୟାନ୍ୟ

9. ଆପଣଙ୍କ ଘରେ ବାସ କରୁଥିବା ଲୋକଙ୍କ ସଂଖ୍ୟା (ଆପଣଙ୍କ ଅନ୍ତର୍ଭୁକ୍ତ କରି)..........

10. ନିଯୁକ୍ତି ସ୍ଥିତି-[_] ନିଯୁକ୍ତିପ୍ରାପ୍ତ[_]ବେକାର[_]ଗୃହିଣୀ[_]ଅବସରପ୍ରାପ୍ତ

11. ଆପଣ ଦୈନିକ ହାରାହାରି କେତେଘଣ୍ଟା କାମ କରିଥାଆନ୍ତି ....(ଘଣ୍ଟା)

12. ସମୁଦାୟ ମାସିକ ଆୟ .... [_] <୧୦ ହଜାର   [_] ୧୦-୨୦ ହଜାର [_] ୨୦-୩୦ ହଜାର [_] > ୩୦ ହଜାର ଟଙ୍କା

13. ଆପଣଙ୍କ ଗୃହର ସମୁଦାୟ ମାସିକ ଆୟ ....ଟଙ୍କା

14. ଏପିଏଲ/ବିପିଏଲ୍ (APL/BPL) (ରେସନ କାର୍ଡ ଅନୁଯାୟୀ)...................................

15. ଗୃହର ପ୍ରକାର [_]କଚ୍ଚା [_]ପକ୍କା[_]ଅର୍ଦ୍ଧ ପକ୍କା

16. ବାସସ୍ଥାନ [_] ସହର [_] ଅର୍ଦ୍ଧ ସହର [_]ଗ୍ରାମାଞ୍ଚଳ

17. ଆପଣ ଜୀବନର ଅଧିକାଂଶ ସମୟ କେଉଁଠାରେ ବିତାଇଛନ୍ତି [_] ସହର [_] ଅର୍ଦ୍ଧ ସହର [_]ଗ୍ରାମାଞ୍ଚଳ

18. କେଉଁ ତାରିଖରେ ଆପଣ ଡାଇବେଟିସ୍ ମେଲିଟସ ରୋଗରେ ଆକ୍ରାନ୍ତ ବୋଲି ଚିହ୍ନଟ କରାଗଲା   __/_ _/ _ _

19. ପ୍ରଥମେ କେଉଁଠାରେ ଆପଣ ଡାଇବେଟିସରେ ଆକ୍ରାନ୍ତ ବୋଲି ଚିହ୍ନଟ କରାଗଲା[_]ସାର୍ବଜନୀନ ସ୍ୱାସ୍ଥ୍ୟ ସେବା ବ୍ୟବସ୍ଥା [_] ଘରୋଇ ସ୍ୱାସ୍ଥ୍ୟସେବା ବ୍ୟବସ୍ଥା

20. ବର୍ତ୍ତମାନ ଆପଣ ଡାଇବେଟିସ ପାଇଁ କୌଣସି ପରାମର୍ଶ ଦିଆଯାଇଥିବା ଔଷଧ ସେବନ କରୁଛନ୍ତି କି [_]ହଁ [_]ନା.

21. ଯଦି ହଁ ଆପଣ ସେବନ କରୁଛନ୍ତି [_]ପାଟିବାଟେ ଖିଆଯାଉଥିବା ମଧୁମେହ ପ୍ରତିହତକାରୀ ବଟିକା [_] ଇନ୍ସୁଲିନ୍ [_] ଉଭୟ

22. ଆପଣ ମଧୁମେହ ପାଇଁ କୌଣସି ବିକଳ୍ପ ଔଷଧ ନେଉଛନ୍ତି କି [_]ଆୟୁର୍ବେଦିକ୍ [_]ହୋମିଓପ୍ୟାଥିକ [_]ଅନ୍ୟାନ୍ୟ [_]କୌଣସିଟି ନୁହେଁ

23. ଏହା ସହିତ ରହିଥିବା ଅନ୍ୟାନ୍ୟ ସ୍ଥିତି		ହଁ / ନା
ଆର୍ଥ୍ରାଇଟିସ୍	A. ଡାକ୍ତରଙ୍କ ଦ୍ୱାରା ଆପଣଙ୍କଠାରେ ***ଆର୍ଥ୍ରାଇଟିସ୍*** **ଚିହ୍ନଟ କରା ଯାଇଛି କି ?**	ହଁ [_] ନା [_]
	ଗତ **12 ମାସ ମଧ୍ୟରେ** ଆପଣ କୌଣସି ଆଘାତ ସମ୍ପର୍କିତ ହୋଇ ନଥିବା ଏକ ମାସରୁ ଅଧିକ ଦିନ ରହିଥିବା **ବ୍ୟଥା, ବିନ୍ଧା, ଗଣ୍ଠି କିମ୍ବା ଗଣ୍ଠି ଚାରିପଟ ଫୁଲିବା ବା ଟାଣ ଲାଗିବା (ଯେପରିକି ହାତ, ବାହୁ, ପାଦ କିମ୍ବା ଗୋଡ)** ***ଅନୁଭବ କରିଛନ୍ତି କି?***	ହଁ [_] ନା [_]
	ଯଦି ହଁ ତେବେ ଆପଣ କୌଣସି ପରାମର୍ଶ ଦିଆଯାଇଥିବା ଔଷଧ ସେବନ କରୁଛନ୍ତି କି?	ହଁ [_] ନା [_]
ଉଚ୍ଚ ରକ୍ତଚାପ	ଆପଣଙ୍କୁ ଜଣେ ଡାକ୍ତରଙ୍କ ଦ୍ୱାରା **ଉଚ୍ଚରକ୍ତ ଚାପରେ ଆକ୍ରାନ୍ତ ବୋଲି ଚିହ୍ନଟ କରାଯାଇଛି କି ?**	ହଁ [_] ନା [_]
	ଯଦି ହଁ ତେବେ ଆପଣ ଉଚ୍ଚ ରକ୍ତ ଚାପ(ହାଇପର୍‌ଟେନ୍‌ସନ୍) ପାଇଁ କୌଣସି ଔଷଧ ସେବନ କରୁଛନ୍ତି କି?	ହଁ [_] ନା [_]
	ପ୍ରଯୁଜ୍ୟ ନୁହେଁ	ପ୍ରଯୁଜ୍ୟ ନୁହେଁ
ପୁରୁଣା ଫୁସ୍‌ଫୁସ୍ ରୋଗ (ଶ୍ୱାସରୋଗ ସହିତ)	ଆପଣଙ୍କଠାରେ କେବେ ବି **ପୁରୁଣା ଫୁସ୍‌ଫୁସ୍** ରୋଗ **(ଏମ୍ଫିଜିମା, ବ୍ରୋଙ୍କାଇଟିସ୍, ଶ୍ୱାସରୋଗ, ସିଓପିଡି) ଚିହ୍ନଟ ହୋଇଛି କି?**	ହଁ [_] ନା [_]
	ଯଦି ହଁ, ଆପଣ ଏଥିପାଇଁ କୌଣସି ପରାମର୍ଶ ଦିଆଯାଇଥିବା ଔଷଧ ସେବନ କରୁଛନ୍ତି କି?	ହଁ [_] ନା [_]
	ପ୍ରଯୁଜ୍ୟ ନୁହେଁ	ପ୍ରଯୁଜ୍ୟ ନୁହେଁ
ଏସିଡ୍-ପେପଟିକ୍ ଡିଜିଜ୍	**ଗତ ୧୨ ମାସ ମଧ୍ୟରେ** ଆପଣଙ୍କଠାରେ ଡାକ୍ତରଙ୍କ ଦ୍ୱାରା **ଏସିଡ୍-ପେପଟିକ୍ ଡିଜିଜ୍** (ଗ୍ୟାସଟ୍ରାଇଟିସ୍) ରୋଗ କେବେ ବି **ଚିହ୍ନଟ** ହୋଇଛି କି ?	ହଁ [_] ନା [_]
	ପ୍ରଯୁଜ୍ୟ ନୁହେଁ	ପ୍ରଯୁଜ୍ୟ ନୁହେଁ
ପୁରୁଣା ଅଣ୍ଟା ବ୍ୟଥା	**ଗତ ୧୨ ମାସ ମଧ୍ୟରେ** ଆପଣଙ୍କଠାରେ ଡାକ୍ତରଙ୍କ ଦ୍ୱାରା **ପୁରୁଣା ଅଣ୍ଟାବ୍ୟଥା** ରୋଗ କେବେ ବି **ଚିହ୍ନଟ** ହୋଇଛି କି ?	ହଁ [_] ନା [_]
	**ଗତ ୧୨ ମାସ ମଧ୍ୟରେ** ଆପଣଙ୍କର ୩ ସପ୍ତାହରୁ ଅଧିକ ଦିନଧରି କ୍ରମାଗତ **ପୁରୁଣା ଅଣ୍ଟା ବ୍ୟଥା** ରୋଗ ହୋଇଛି କି ?	ହଁ [_] ନା [_]
ହୃଦରୋଗ	**ଗତ ୧୨ ମାସ ମଧ୍ୟରେ** ଆପଣଙ୍କଠାରେ ଡାକ୍ତରଙ୍କ ଦ୍ୱାରା **ଆଞ୍ଜାଇନା ହାର୍ଟ ଆଟାକ୍/ହୃଦରୋଗ** କେବେ ବି **ଚିହ୍ନଟ** ହୋଇଛି କି ?	ହଁ [_] ନା [_]
	ଯଦି ହଁ, ଆପଣ ଏଥିପାଇଁ କୌଣସି ପରାମର୍ଶ ଦିଆଯାଇଥିବା ଔଷଧ ସେବନ କରୁଛନ୍ତି	ହଁ [_] ନା [_]
	**ଗତ ୧୨ ମାସ ମଧ୍ୟରେ** ଆପଣଙ୍କ କେବେ ବି ପାହାଡ ଚଢ଼ିବା ବେଳେ ବା ଦରକାର ହୋଇ ଚାଲିଲା ବେଳେ ବା ସାଧାରଣ ଚାଲିବା ସମୟରେ ଛାତିରେ କୌଣସି ପ୍ରକାରର ***ଅସହଜତାବୋଧ ବା ବ୍ୟଥା*** ଅନୁଭବ କରିଛନ୍ତି କି ?	ହଁ [_] ନା [_]

ଷ୍ଟ୍ରୋକ୍	ଆପଣଙ୍କୁ କେବେ କୌଣସି ସ୍ୱାସ୍ଥ୍ୟ ପେସାଦାର **କହିଛନ୍ତି ଯେ,** ଆପଣଙ୍କର **ଷ୍ଟ୍ରୋକ୍** ହୋଇଥଲା?	ହଁ [_] ନା [_]
	ଯଦି ହଁ, ଆପଣ ଏଥିପାଇଁ କୌଣସି ପରାମର୍ଶ ଦିଆଯାଇଥିବା ଔଷଧ ସେବନ କରୁଛନ୍ତି କି?	ହଁ [_] ନା [_]
	**ଗତ ୧୨ ମାସ ମଧ୍ୟରେ** ଆପଣଙ୍କ କେବେ ବି ହଠାତ୍ **ପକ୍ଷାଘାତ** ଦେଖାଦେବା ଆପଣଙ୍କ ଶରୀରର ଗୋଟିଏ ପାର୍ଶ୍ବର ହାତ ବା ଗୋଡ ୨୪ ଘଣ୍ଟାରୁ ଅଧିକ ସମୟ **ଦୁର୍ବଳ ଅନୁଭବ** ହେବାରେ ଆକ୍ରାନ୍ତ ହୋଇଛନ୍ତି କି?	ହଁ [_] ନା [_]
ଦୃଷ୍ଟିହୀନତା	ଆପଣ ଡାକ୍ତରଙ୍କ ଦ୍ୱାରା **ଦୃଷ୍ଟିହୀନତାରେ** ଆକ୍ରାନ୍ତ ବୋଲି **ଚିହ୍ନଟ** ହୋଇଛନ୍ତି କି?	ହଁ [_] ନା [_]
	ଆପଣଙ୍କର କୌଣସି ଦୃଷ୍ଟିଦୋଷ ରହିଛି କି? (ଯଦି ଆପଣ ଚଷମା ପିନ୍ଧି ଭଲ ଭାବରେ ଦେଖି ପାରନ୍ତି ତେବେ ଉତ୍ତରରେ ନା କହନ୍ତୁ)	ହଁ [_] ନା [_]
ବଧିରତା	**ଗତ ୧୨ ମାସ ମଧ୍ୟରେ** ଆପଣଙ୍କଠାରେ ଡାକ୍ତରଙ୍କ ଦ୍ୱାରା **ବଧିରତା** କେବେ ବି **ଚିହ୍ନଟ** ହୋଇଛି କି ?	ହଁ [_] ନା [_]
	**ଗତ ୧୨ ମାସ ମଧ୍ୟରେ** ଆପଣଙ୍କଠାରେ ଡାକ୍ତରଙ୍କ ଦ୍ୱାରା **ବଧିରତା ବା ୩ ମାସରୁ ଅଧିକ ସମୟ ପର୍ଯ୍ୟନ୍ତ ଶୁଣିବାରେ ଅସୁବିଧା ହେବା** କେବେ ବି **ଚିହ୍ନଟ** ହୋଇଛି କି ?	ହଁ [_] ନା [_]
କର୍କଟ	ଆପଣଙ୍କଠାରେ ଡାକ୍ତରଙ୍କ ଦ୍ୱାରା **କୌଣସି ପ୍ରକାରର କର୍କଟ** ରୋଗ କେବେ ବି **ଚିହ୍ନଟ** ହୋଇଛି କି ?	ହଁ [_] ନା [_]
ଦୀର୍ଘଦିନର ବୃକକ୍ ସମସ	ଆପଣଙ୍କଠାରେ ଡାକ୍ତରଙ୍କ ଦ୍ୱାରା **ଦୀର୍ଘଦିନର ବୃକକ୍ ସମସ୍ୟା** କେବେ ବି **ଚିହ୍ନଟ** ହୋଇଛି କି ?	ହଁ [_] ନା [_]
	ଆପଣଙ୍କଠାରେ କେବେ ବି ଡାଇଲିସିସ୍ ହୋଇଛି କି?	ହଁ [_] ନା [_]
ଅପସ୍ମାର	କେବେ କୌଣସି ସ୍ୱାସ୍ଥ୍ୟ ପେସାଦାର ଆପଣଙ୍କର **ଅପସ୍ମାର** ରହିଛି ବୋଲି **କହିଛନ୍ତି କି**?	ହଁ [_] ନା [_]
	ଯଦି ହଁ, ଆପଣ ଏଥିପାଇଁ କୌଣସି ପରାମର୍ଶ ଦିଆଯାଇଥିବା ଔଷଧ ସେବନ କରୁଛନ୍ତି କି ?	ହଁ [_] ନା [_]
	ଆପଣ *କାମ କରୁଥିବା ସମୟରେ ବା ବିଶ୍ରାମ ନେଉଥିବା ସମୟରେ* କେବେ ବି **ହଠାତ୍ ବାତ ମାରିବାରେ** ଆକ୍ରାନ୍ତ ହୋଇଛନ୍ତି କି?	ହଁ [_] ନା [_]
ଥାଇରଏଡ୍ ରୋଗ	ଆପଣଙ୍କଠାରେ ଡାକ୍ତରଙ୍କ ଦ୍ୱାରା କେବେ ବି **ଥାଇରଏଡ୍ ରୋଗ ଚିହ୍ନଟ** ହୋଇଛି କି?	ହଁ [_] ନା [_]
	ଯଦି ହଁ, ଆପଣ ଏଥିପାଇଁ କୌଣସି ପରାମର୍ଶ ଦିଆଯାଇଥିବା ଔଷଧ ସେବନ କରୁଛନ୍ତି କି ?	ହଁ [_] ନା [_]
ଟ୍ୟୁବରକୁଲୋସିସ୍ ବା ରାଜଯକ୍ଷ୍ମା	ଆପଣ ***ଟିବି***ରେ ଆକ୍ରାନ୍ତ କି?	ହଁ [_] ନା [_]
	ଆପଣ ***ଟିବି*** ପାଇଁ କୌଣସି ଚିକିତ୍ସା ନେଉଛନ୍ତି କି?	ହଁ [_] ନା [_]
ବିଷାଦ	ଡାକ୍ତରଙ୍କ ଦ୍ୱାରା ଆପଣଙ୍କଠାରେ କେବେ ବି ବିଷାଦ **ଚିହ୍ନଟ** କରାଯାଇଛି କି?	ହଁ [_] ନା [_]
	**ଗତ ୧୨ ମାସ ମଧ୍ୟରେ** ଆପଣ **ଦୁଃଖିତ, ବିଷାଦଗ୍ରସ୍ତ, ଚିନ୍ତାଗ୍ରସ୍ତ କିମ୍ବା ଉଦ୍‌ବେଗ ଅନୁଭବ କରିବା** କାରଣରୁ ଡାକ୍ତରଙ୍କ ସହିତ ପରାମର୍ଶ କରିଛନ୍ତି କି?	ହଁ [_] ନା [_]
	ଯଦି ହଁ, ଆପଣ ବିଷାଦ ପାଇଁ କୌଣସି ପରାମର୍ଶ ଦିଆଯାଇଥିବା ଔଷଧ ସେବନ କରୁଛନ୍ତି କି ?	ହଁ [_] ନା [_]
	ପ୍ରଯୁଜ୍ୟ ନୁହେଁ	ପ୍ରଯୁଜ୍ୟ ନୁହେଁ

24. ଆପଣ ଏହି ପୁରୁଣା ସ୍ୱାସ୍ଥ୍ୟ ସମସ୍ୟାଗୁଡିକ ମଧ୍ୟରୁ କୌଣସିଟିରେ ଆକ୍ରାନ୍ତ କି? [_] ହଁ [_] ନା

ଯଦି ହଁ, ସେଗୁଡିକର ନାମ ଲେଖନ୍ତୁ -

1...

- ଏହା ଡାକ୍ତରଙ୍କ ଦ୍ୱାରା ଚିହ୍ନଟ କରାଯାଇଥିଲା କି? [_] ହଁ [_] ନା

- ଆପଣ ଏଥିପାଇଁ କୌଣସି ପରାମର୍ଶ ଦିଆଯାଇଥିବା ଔଷଧ ସେବନ କରୁଛନ୍ତି କି ? [_] ହଁ [_] ନା

- ଏହା ଆପଣଙ୍କ ଦୈନନ୍ଦିନ କାର୍ଯ୍ୟକଳାପକୁ କେତେ ପରିମାଣରେ ସୀମିତ କରୁଛି?
1. ଆଦୌ ନୁହେଁ [_] 2.ସାମାନ୍ୟ [_] 3.କେତେକାଂଶରେ [_] 4. ଯଥେଷ୍ଟ ପରିମାଣରେ [_] 5. ବହୁ ପରିମାଣରେ [_]

2...

- ଏହା ଡାକ୍ତରଙ୍କ ଦ୍ୱାରା ଚିହ୍ନଟ କରାଯାଇଥିଲା କି? [_] ହଁ [_] ନା
- ଆପଣ ଏଥିପାଇଁ କୌଣସି ପରାମର୍ଶ ଦିଆଯାଇଥିବା ଔଷଧ ସେବନ କରୁଛନ୍ତି କି ? [_] ହଁ [_] ନା

- ଏହା ଆପଣଙ୍କ ଦୈନନ୍ଦିନ କାର୍ଯ୍ୟକଳାପକୁ କେତେ ପରିମାଣରେ ସୀମିତ କରୁଛି ?
1. ଆଦୌ ନୁହେଁ [_] 2.ସାମାନ୍ୟ [_] 3.କେତେକାଂଶରେ [_] 4. ଯଥେଷ୍ଟ ପରିମାଣରେ [_] 5. ବହୁ ପରିମାଣରେ [_]

25. ଆପଣ ମାସିକ ଔଷଧ ପାଇଁ କେତେ ଖର୍ଚ୍ଚ କରିଥାନ୍ତି -?

1. ଡାଇବେଟିସ୍ ପାଇଁ-...............ଟଙ୍କା

2. ଅନ୍ୟାନ୍ୟ ରୋଗ ପାଇଁ.............ଟଙ୍କା

26. ଆପଣ ଡାକ୍ତର ବା ସ୍ୱାସ୍ଥ୍ୟକେନ୍ଦ୍ରକୁ ଯାତାୟତ କରିବା ପାଇଁ ପ୍ରତ୍ୟକ ସାକ୍ଷାତ ପିଛା କେତେ ଟଙ୍କା ଖର୍ଚ୍ଚ କରିଥାନ୍ତି ....ଟଙ୍କା

27. ଆପଣ ଗତ ୬ ମାସ ମଧ୍ୟରେ ଏକ ସ୍ୱାସ୍ଥ୍ୟକେନ୍ଦ୍ର ବ୍ୟବସ୍ଥାକୁ କେତେଥର ଯାଇଛନ୍ତି ? ....

28. ଆପଣ ୬ ମାସ ମଧ୍ୟରେ ହାରାହାରି କେତେ ଟଙ୍କା ଡାଇବେଟିସ ପାଇଁ ପରୀକ୍ଷାଗାର ପରୀକ୍ଷା ପାଇଁ ଖର୍ଚ୍ଚ କରିଛନ୍ତି (ରକ୍ତ ସର୍କରା ପରୀକ୍ଷା, ଗ୍ଲାଇସେଟେଡ୍ ହିମୋଗ୍ଲୋବିନ ଆକଳନ) ?...ଟଙ୍କା

- ଆପଣ ଏହି ପରୀକ୍ଷା ପାଇଁ କେତେ ସମୟ ବିତାଇ ଥାନ୍ତି? ....

29. ଆପଣ ଅନ୍ୟ ପୁରୁଣା ରୋଗ ପାଇଁ ପରୀକ୍ଷାଗାର ପରୀକ୍ଷା ବାବଦକୁ କେତେ ଟଙ୍କା ଖର୍ଚ୍ଚ କରିଥାଆନ୍ତି? ....ଟଙ୍କା

- ଆପଣ ଏହି ପରୀକ୍ଷା ପାଇଁ କେତେ ସମୟ ବିତାଇ ଥାଆନ୍ତି?......................................

30. ଉଚ୍ଚତା.................................

ଓଜନ.................................

ବିଏମ୍ଆଇ........................... > 25 ହଁ [_] ନା [_]

< 25 ହଁ [_] ନା [_]
